# Exploring Influential Factors Including COVID-19 on Green Food Purchase Intentions and the Intention–Behaviour Gap: A Qualitative Study among Consumers in a Chinese Context

**DOI:** 10.3390/ijerph17197106

**Published:** 2020-09-28

**Authors:** Xin Qi, Huaming Yu, Angelika Ploeger

**Affiliations:** 1 Specialized Partnerships in Sustainable Food Systems and Food Sovereignty, University of Kassel, 37213 Kassel, Germany; a.ploeger@uni-kassel.de; 2 Sanya Oceanographic Institution, Ocean University of China, Sanya 572024, China; hmyu@ouc.edu.cn; 3 Qingdao National Laboratory of Marine Science and Technology Co., Ltd., Qingdao 266237, China

**Keywords:** green food consumption, purchase intention, intention–behaviour gap, COVID-19, qualitative method, China

## Abstract

This study applied a qualitative approach to investigate the underlying influences on consumers’ green food consumption from the intention generation phase to intention execution phase in the perspectives of purchase intention and the intention–behaviour gap (IBG). Additionally, the impact of the “Coronavirus Disease 2019” (COVID-19) pandemic on consumers’ green food purchases was explored. Research data were derived from semi-structured in-depth interviews with 28 consumers and analyzed using grounded theory. The findings identified factors that influenced intentions and the IBG in the process of consumers’ green food purchases. Specifically, these findings reported that health consciousness, perceived attributes, environmental consciousness, social influence, family structure, and enjoyable shopping experiences were identified as major drivers for generating consumers’ green food purchase intentions. High prices of green food, unavailability issues, mistrust issues, and limited knowledge were factors triggering the gap between green food purchase intentions and behaviours. In addition, the results revealed that the COVID-19 crisis increased consumers’ green food purchase intentions, whereas the IBG widens as a result of issues of unavailability, price, and panic. These findings will help stakeholders build future policy and suitable strategies to better promote green food consumption in the Chinese context.

## 1. Introduction

With the exploitation and destruction of the environment and natural resources, the concept of “green consumerism” has prospered and attracted increasing attention in the world [1,2]. Food consumption has been seen as a major issue of achieving sustainability, because it is associated with the environment, individual and public health, social cohesion, and the economy [3]. As the world’s largest food consumer group, Chinese consumers have profoundly changed their consumption patterns and increasingly started preferring environmentally friendly food products after a series of environmental problems (e.g., poor air quality, heavy smog) and frequent food safety scandals (e.g., the gutter oil scandal, abuse of sodium cyclamate) [4,5]. Facing this tendency, the Chinese government has adopted the green food certification system to improve the environment and ensure public health [6]. Green food refers to the safe and premium edible agricultural products and related processed products that are grown in an ecologically sound environment, are produced according to the green food production standard, adopt the whole-some quality control, and grant the right to use the “green food” logo [7]. There are two standards for green food: Grade A and Grade AA. Grade A represents a transitional level between conventional and organic food, allowing to use a certain amount of chemical materials, and Grade AA is equivalent to international organic standards [5]. During the past five years, the Chinese green food sector has been displaying a flourishing vigour and has been growing at an average of 9.2% domestic sales [7]. In the year 2018, there were a total of 30,932 green food labelled products, 106,523 thousand tons of green food production, and 68.1 billion U.S. dollars of total domestic sales [7]. Despite the growth in the green food industry, green food market and consumer expenditure shares of green food products are extremely low, which constitute only less than 1% of total Chinese food sales in 2018 [8]. Therefore, the green food sector shows great potential for further expansion in China.

To effectively respond to the growth of the green food market, it is important to study its consumers’ purchasing behaviours, especially focusing on purchase intention, which is the premise of performed purchase behaviour [9]. Thus, marketers and academicians need to deeply understand which factors motivate consumers’ intentions to purchase green food in China. However, research related to consumer intentional behaviour towards green food in the Chinese context is still insufficient, as most of the studies have been focused on organic food products [10,11,12,13,14,15,16]. For example, Liu et al. [6] found the specific attributes of safety, health, nutrition, and taste as the key drivers of organic food preferences; among them, safety was the most important factor for consumers to generate buying intentions. The study from McCarthy et al. [17] reported that purchasing intentions of organic food were motivated by altruistic concerns (i.e., environment and animal welfare concerns) and self-interest (i.e., personal and familial health concerns, food safety concerns). In terms of the green food consumption issue, Wang and Wang [18] and Zhu et al. [19] applied the theory of planned behaviour (TPB) to study the influence mechanisms in the process of green food purchases, and the TPB constructs (i.e., attitude, subjective norm, perceived behavioural control) were found to have significant effects. Several studies have determined personal characteristic factors (e.g., gender, age, family size, education, income) that influence green food purchase behaviour [5,19]. Although prior studies have provided some evidence on the factors of organic and green food consumption, researchers still suggest to verify their impacts and explore additional factors in further research [6,20].

Moreover, the upward trend and low market share also can be caused by a phenomenon called the intention–behaviour gap (IBG), whereby a highly positive intention towards behaviour is expressed, followed by not acting in the depicted way owing to different reasons [21,22]. The green consumption, which consumers have increasingly perceived as environmentally responsible behaviour, is considered as a strong representation of this gap [22]. For example, some surveys reported that 30%–50% of consumers have expressed their intentions to purchase sustainable products, whereas only 5% of consumers have completed their actual buying behaviours [23,24]. This inconsistent phenomenon is also confirmed by other green studies [25,26,27], whereas most studies have investigated the IBG in the fields of pro-environmental manner and organic food consumption instead of green food. Although the results of organic food can be used for comparison and reference [5], they still need to be examined and validated in the domain of green food consumption. Moreover, many researchers have argued that the green IBG is still unexplained because of inconclusive results and the lack of systematic research [22]. Subsequently, researchers call for further investigations to help minimize the gap by exploring factors that hinder consumers from translating their intention into practice [24,25].

Furthermore, the emergence of coronavirus disease 2019 (COVID-19) has significantly affected the global food systems of producers, processors, and consumers, at different levels [28,29]. Notably, the public’s hygiene behaviours [30] and consumption patterns have shifted as the coronavirus pandemic has progressed [31,32,33]. The latest study from Ben Hassen et al. [33] reported that the COVID-19 pandemic could shift consumers’ behaviour in a more sustainable and healthier direction and increase the consumption of local food products because of food safety concerns. Moreover, the results from Xie et al. [28] revealed that the COVID-19 crisis influenced the respondents’ perceptions and attitudes towards organic food thanks to its safer and healthier characteristics, which can lead to a change in consumers’ future diets. As this infectious disease is new and its lifespan is not known, there is a need to gather more data and information to explore COVID-19 impacts on shifting food consumption patterns. Therefore, considering the safer, healthier, and sustainable characteristics of green food products, there is a critical need to study changes in Chinese consumers’ attitudes and behaviours towards green food to address the potential for long-term health consequences due to behaviour changes during the pandemic. Although some researchers have studied factors that influence purchase intentions and the IBG in green food consumption, the majority of previous studies applied quantitative methods with close-ended questions, which may limit participants in expressing their true motivations, barriers, and perceptions of buying green food products [34]. However, according to Cohen [35], qualitative research with consumers can be more systematic, psychological, and innovative, which can reveal the “why” behind consumer behaviour and may explore underlying motives, values, and attitudes towards a particular product. Specifically, the latest research from ElHaffar et al. [36] has highlighted the importance of qualitative studies and experimental designs in closing the IBG in green consumption. In addition, studying the influence of COVID-19 on consumers’ behaviour is still at a nascent stage. None of the studies have measured green food consumption with a special emphasis on COVID-19 influences in the Chinese context. When considering the continuous involvement in consumers’ lifestyles and consumption patterns, qualitative research with open-ended, discovery-oriented methods is needed to explore green food consumption changes of Chinese consumers during a detrimental global pandemic. Thus, the present study has examined the underlying influences on consumers’ green food consumption from the intention generation phase to the intention execution phase in a Chinese context using a qualitative inquiry.

The three main aims of this study are (i) to explore which factors motivate the intention of consumers to purchase green food, (ii) to investigate which factors prevent consumers from translating their intention into green food purchase behaviour, and (iii) to study the impacts from the COVID-19 crisis for consumers on green food consumption. Our study is based on grounded theory by employing in-depth interviews. The outcome of this research has significant practical implications for different stakeholders including scholars, green food marketers, and policymakers. The research findings can enable stakeholders to comprehend the important role of the IBG and the mechanisms that influence green food consumption of consumers. Furthermore, this study can provide newer insight into the impact of the pandemic crisis on consumers’ green food purchases. Therefore, governments and stakeholders of the green food industry can utilize these findings to develop appropriate public health and marketing strategies to promote healthier lifestyles and green food consumption in the future.

## 2. Methods

### 2.1. Design

A qualitative study was conducted using semi-structured in-depth interviews and grounded theory. Initially, this study applied semi-structured in-depth interviews with the aid of probing questions to collect data, which can provide richer insights into complex phenomenon from an open-ended and discovery-oriented perspective [37]. Furthermore, grounded theory was used to analyze the data in our study. According to Goulding [38], grounded theory enables researchers to generate theories that “(i) enable an explanation of behaviour, (ii) are useful in advancing theory, (iii) are applicable in practice, (iv) provide a perspective on behaviour, (v) guide and provide a style for research on particular areas of behaviour, and (vi) provide clear enough categories and hypotheses that crucial ones can be verified in the present and future research”. This scientific method assists the researcher to keep an open mind in the detection and explanation of social phenomena from respondents by answering socially purposeful questions [38,39]. Semi-structured interviews are consistent with grounded theory because it allows the researcher to pose key questions, consecutively in the same fashion, but allows flexibility in the sequencing of questions and the depth of exploration [37]. This study followed the Straussian approach by designing preliminary interview questions in a strategic way, which did not violate basic theoretical principles. Then, new items were added to the preliminary questions guide and researchers continued to interview consumers for data collection until additional responses stopped generating any further insights into the questions [40]. The recursive process included data collection, data coding, comparative analysis, and theoretical sampling until data saturation [38,40].

### 2.2. Participates and Procedure 

The target group of our study is consumers who have purchased green food in the past. A theoretical sampling technique was used for selecting samples in this study [41]. The recruitment information was spread via WeChat, the most popular instant messaging mobile application in China, where respondents can attend an interview through their mobile phones. To stimulate people’s willingness to participate in the interview, the participants can receive 50 RMB of “red packets”, an electronic monetary payment on WeChat. The proposed time of the interview was approximately 25–30 min. Those who agreed to participate were asked to provide their contact information and the researcher would discuss with respondents to arrange an interview appointment. Before starting the interview, each respondent was asked whether they have ever purchased green food products, and respondents were requested to continue with the interview only if their answer was positive. There were six basic topics to be discussed during the interview: (i) green food purchase experience, (ii) general thoughts about green food, (iii) factors that drive green food purchase intention, (iv) the green IBG, (v) factors triggering the IBG, and (vi) the impact of COVID-19 on green food purchase intention and the IBG. The questions in the interview guide were borrowed from past studies and modified for this study. Then, the interview guide was back-translated by native speakers to confirm the correct answers. All interviews were recorded and transcribed. Participants were informed that their responses would be audio recorded and that their data would be anonymized and stored securely. Two researchers coded interviews independently to ensure the reliability, and quality control checks were conducted to validate all interview summaries. For member checks, this study contacted several interviewees for the second time, in order to examine whether the researchers’ interpretations of the respondent’s experience were presented correctly or not. The data collection process lasted for 20 days in May 2020. Finally, a total of 28 consumers participated in this investigation and data saturation was achieved after the 28th participant, as no new codes were being generated from the new respondents. Descriptive information about the respondents’ characteristics is provided in Table 1.

### 2.3. Data Analysis

For questions such as asking respondents’ experience, descriptive statistics were used to analyze the results. For the questions investigating consumers’ thoughts about green food, the number count for each word was tallied and words with similar meanings were grouped into categories and dimensions. With regard to measuring the purchase frequency, respondents were categorized into three groups: (i) regular buyers (i.e., consumers buy more than green food), (ii) irregular buyers (i.e., buy green and other food equally), and (iii) casual buyers (i.e., buy other food more), based on the categorization used by Rana and Paul [42]. For qualitative questions such as exploring factors that drive green food purchase intention and trigger IBG, grounded theory was used to conduct data coding [39], which followed the procedure from Yadav et al. [43]. Data coding was analyzed in different phases: (i) open coding (i.e., identifying recurring patterns in the responses), (ii) axial coding (i.e., merging the closely related open codes under broader dimensions), and (iii) selective coding (i.e., looking for connections and statements) [39]. When analyzing each paragraph, the audio transcript of every participant was divided into a set of paragraphs. Furthermore, the identification of statements and connections for each paragraph was tracked and checked against the literature [39]. The integration and analyses of all data took place in the final phase.

## 3. Results

### 3.1. Experiences and Thoughts of Green Food Purchase

Regarding the results of the last purchase experience of green food, 32.14% of respondents indicated that they had purchased green food products in the last week, 39.29% had bought green food products in the last month, and 28.57% had purchased green food more than one month ago. In terms of probing which latest green food products they purchased, vegetables, rice, and vegetable oils were the most common products, which were mentioned 11 times, 6 times, and 4 times by interviewees, respectively. When respondents were asked to describe the first three words that come to their minds about green food, a total of 21 different individual words were recorded. Among these words, ‘’healthy’’ (16), ‘’high price’’ (10), and ‘’safe’’ (8) were the most frequently mentioned words by consumers. All words were summarized and grouped into four dimensions, based on categorization used by Ismael and Ploeger [44]. Table 2 shows the frequency of mention of individual words in four categories.

### 3.2. Factors of Driving Green Food Purchase Intention

On the basis of the analysis of the respondents’ interviews, 16 open codes were identified as factors that drive green food purchase intention. Through merging closely related open codes under broader dimensions, six axial codes were generated as drivers of green food purchase intention, which were health consciousness, perceived attributes, environmental consciousness, social influence, family structure, and enjoyable shopping experience.

#### 3.2.1. Axial Code 1: Health Consciousness

Nowadays, food consumption patterns are rapidly changing, thus a growing number of consumers are starting to choose environmentally friendly food products [4]. Health consciousness among consumers is one of the most motivating factors, especially for environmentally friendly food consumption [10,44,45,46,47]. Health-related topics were also the most frequently mentioned by participants in the study. This axial code of health consciousness consists of three open codes: health concerns; fewer pesticides, chemical fertilizers, and additives; and safety concerns.

- Health Concerns

Many participants reported that health concerns were the most important reason for generating their green food purchase intentions. The participants felt that they would become healthier while eating green food, compared with consuming conventional food. Moreover, they felt that the health benefits offered by green food were greater than its cost, thus they were more likely to buy it.

“I want to purchase green food mainly because of the perceived health benefits of eating green food products. I feel much healthier compared with when consuming conventional food. Although the price of green food is slightly higher than conventional food products, I think it is worth for having better health.” (Participant 2)

- Fewer Pesticides, Chemical Fertilizers, and Additives

Some participants thought green food is a healthier option than conventionally produced food because of its more natural production method, with fewer pesticides and fertilizers. The participants were motivated to consider food made from natural ingredients instead of synthetic and artificial additives.

“There are fewer toxins such as pesticides, chemical fertilizers, and additives in green food products. Therefore, I can consume green food with more reassurance. I am afraid that eating food containing chemicals or additives can harm a person’s health.” (Participant 15)

- Safety Concerns

As a result of recurrent food-safety incidents and scandals in Chinese food markets, several participants were influenced by the importance of food safety during their food purchase process. They believed that green food was healthier and safer, which became a preference.

“Food safety issues have been making me panic, and eating green food makes me feel safer.” (Participant 4)

#### 3.2.2. Axial Code 2: Perceived Attributes

Attributes of food, such as appearance, taste, and nutritional value, are also considered as crucial determinants for consumers making food choices [48,49]. Consumers have perceived both functional and ethical attributes to fulfil their personal needs and desires so that they prefer to choose green products [50,51]. Most participants also reported that functional and sustainable characteristics of products combined with their high quality had positively influenced their green food purchase intentional behaviour. This axial code of perceived attributes is made up of three open codes: nutritious, tasty, and natural; better quality; and good packaging.

- Nutritious, Tasty, and Natural

Some participants perceived that green food products can offer more nutritional value and better flavour. They felt green food products are more natural because of their cultivation and production process.

“I think green food is more nutritious and tasty, especially like the taste of food when I was a kid. Moreover, I feel green food is closer to nature.” (Participant 1)

- Better Quality

Most of the interviewees believed that green food had better quality compared with other conventional food products. Moreover, they preferred high-quality products rather than compromising quality and buying at a low price. Therefore, quality consciousness was also one salient motivator for the participants when considering their green food choice.

“The quality of green food products is obviously better than other food products, so I prefer to choose the better ones.” (Participant 22)

- Good Packaging

The material and appearance of green food packaging were also mentioned by participants, which enhanced their intentions during the food purchasing process.

“Green food products are packaged cleanly with some recyclable and safe materials, which is important to me. Additionally, when sending gifts to leaders and friends, I prefer to choose green food because of its quality packaging.” (Participant 13)

#### 3.2.3. Axial Code 3: Environmental Consciousness

Recently, consumers have increasingly shown their concerns and responsibility towards environmental and sustainable issues, which are found to have a positive influence on green purchase intention [52]. Particularly, environmental consciousness is identified as a very important motivator for environmentally friendly food consumption, which is perceived as one pro-environmental and sustainable behaviour [53,54]. This axial code of environmental consciousness includes two open codes: protect the environment and sustainability.

- Protect the Environment

Some interviewees generally related green food to environmentally friendly planting and manufacturing processes. They believed the method of producing green food was healthy for the environment and society, thus they had positive attitudes towards green food.

“I think growing and producing green food is helpful to protect the environment and society, thus I would like to make my contribution to protect the environment through consuming green food.” (Participant 6)

- Sustainability

Several participants discussed the sustainable impact of purchasing green food behaviour, and they emphasized the benefits of sustainability for future development.

“Consuming green food is a sustainable behaviour, and I think it also can bring benefits for future generations.” (Participant 10)

#### 3.2.4. Axial Code 4: Social Influence

Even if food consumption is a personal choice, social influence still plays a salient role in consumption patterns, especially in a Chinese context with typical collectivistic characteristics [16,18,55]. Some studies revealed that green purchase intention behaviour is associated with social norms and values, social groups, and cultural influences [18,56]. Consumers follow social norms to receive social acceptance in their groups and to avoid losses from mistaken decisions. Gaining status is an important reason for choosing green or high-quality products [18,57]. This study retrieved statements from participants from the perspective of social influence. This axial code is composed of three open codes: trend, group conformity, and gain status.

- Trend

Participants indicated that it is a trend to pursue a healthy and green life, especially when they perceived this trend from their friends and some celebrities in their social networks.

“Buying green and healthy food is a social trend now. My friends and some celebrities like to post their green lifestyles on the Internet and promote the benefits of eating green, which motivates me to follow this green trend.” (Participant 28)

- Group Conformity

Group influences were mentioned by interviewees in that group orientation had a significant positive effect on their green food purchase intentions, and they choose to comply with recommendations from their acquaintances or reference groups.

“I prefer to choose green food products, especially when friends and colleagues recommend or buy them.” (Participant 7)

- Gain Status

Individuals in the study also perceived an enhanced image and status in their social system when buying green food. They felt they gained “face” in their social activities, which motivated them to pay greater attention to green food products.

“I would be noticed as a higher status by my peers and I can gain ‘face’ if I purchase green food.” (Participant 23)

#### 3.2.5. Axial Code 5: Family Composition

Family size and composition are important socio-cultural factors influencing consumers’ intentions and behaviours [6,58]. Families with infants, children, or elders are more inclined to buy safe or green food products [6,59]. Many respondents with older parents and children mentioned this effect in our study. Although some respondents also expressed their health concerns for their family members, the responses still reflected that their intentions are motivated by their concerns for their family members and structure. Therefore, this axial code of family composition is identified by two open codes: buy for children and buy for the elderly.

- Buy for Children

Most of the interviewees as parents reported that they were very concerned about their children’s growth and preferred to purchase green food for them.

“The main reason that I intend to buy green food is for my son. I want him to eat safe and healthy food, which will help him grow up better.” (Participant 8)

- Buy for the Elderly

Some participants with elders expressed their purchase demand for green food because of their consideration for their parents’ health and life.

“My parents are now in their 80s, and I would like to buy them green food.” (Participant 6)

#### 3.2.6. Axial Code 6: Enjoyable Shopping Experience

With consideration of special properties of green food, some companies have tailored marketing strategies accordingly to attract consumers’ interests and promote their sales, such as well-trained salesmen, enjoyable shopping environments, and attractive promotion plans. These marketing strategies also play an important role in deciding consumers’ willingness to pay [6]. Some participants reported that they normally had an enjoyable shopping experience during the process of buying green food and it results in a positive impression. This axial code is made from three open codes: better attitudes from a salesman, better shopping ambiance, and promotion.

- Better Attitudes from A Salesman

Several interviewees mentioned that sales employees at the green food counter were generally enthusiastic and provided good service, which resulted in the willingness to buy green food.

“The attitude of salesmen at the green food counter is generally very good, and they usually give me a good introduction and suggestions.” (Participant 22)

- Better Shopping Ambiance

Some interviewees described their previous shopping environment when buying green food, which was clean, comfortable, well-decorated, and upscale.

“The green food sales area is classy and high-grade and it provides for a positive shopping experience so that I am more eager to shop.” (Participant 1)

- Promotion

Some participants expressed that the sale promotion activities strongly motivated their intentions to buy green food, which made their shopping a bargain.

“I often buy green food products, if there are some promotions, such as buying three get one free and buying two get one free.” (Participant 8)

### 3.3. IBG in Green Food Purchase

The consumers’ IBG is one of the most important issues facing the green food market. Thus, the present study has measured the gap in participants’ green food purchases and the disparity between the percentage of buying green food in their daily life and desired green purchases in the case of the absence of factors pertaining to the gap. Most of the participants had experienced a gap between what they planned and what they purchased, and they also reported that their green food purchases would increase if the obstacles they faced (e.g., high price, unavailability) would disappear. Green food purchase frequency was measured by asking consumers about the percentage of green food purchases from their entire food purchases. According to the categorization used by Rana and Paul [42], respondents were categorized into three groups of green food consumers, which are presented in Table 3. In terms of green food purchase behaviour, 8 respondents were grouped into regular buyers, 4 respondents were irregular buyers, and 16 respondents belonged to causal buyers. Regarding green food purchase intention, 17 respondents were grouped into regular buyers, 5 respondents were irregular buyers, and 6 respondents belonged to causal buyers. The percentage of regular buyers would increase from 28.57% to 60.71%, the percentage of irregular buyers would increase from 14.28% to 17.86%, and the percentage of casual buyers would decrease from 57.15% into 21.43% (Table 3). This discrepancy between consumers’ daily behaviour and their intentions to purchase green food represents the IBG in their green food consumption. Participants have questioned the possible hindrances behind this gap scenario, which will be explained in detail in Section 3.4.

### 3.4. Factors of Triggering the IBG

In terms of factors preventing consumers from translating their intention into green food purchase behaviour, eight open codes were identified from the participants. Through reanalyzing the data, four common themes for axial codes were formed, which were high price, unavailability issues, mistrust issues, and limited knowledge.

#### 3.4.1. Axial Code 1: High Price

Higher prices are reported to be one main barrier for consumers to transform their intentions into the final purchasing of environmentally sustainable products, because most of the consumers perceive the price as premium and addressed to a niche segment [25,60,61]. Consumers are usually sensitive towards price, and they are willing to buy eco-friendly food products, but not at higher prices [21]. Even for regular buyers of eco-friendly food products, the most important reason for not purchasing these products was high pricing issues [62]; 18 participants in our investigation admitted that they were very price sensitive and reported that price was a major concern when they wanted to buy green food.

- High Price

Price barrier was described as interviewees least willing to pay a higher price for a green food product. Moreover, some participants also mentioned the problem of inflation and affordability, which limited their expenses.

“I know about the benefits of green food, but some products are just too expensive, almost double the price of conventional products. Now the price of necessities is rising and I could not afford to buy all food at higher prices.” (Participant 13)

#### 3.4.2. Axial Code 2: Unavailability Issues

Limited availability and difficulty in accessing environmentally sustainable products are identified as major factors triggering the IBG [6,25,63]. Some consumers believe green food is not easily available in most of the supermarkets, and they are unwilling to spend more time in procuring environmentally sustainable products [64]. Thus, the structures of the distribution can be seen as a key factor to limit demand and behaviour. Even if available, there are few varieties of green food products in the market. These issues were also discussed by participants in the interviews, which form the axial code of unavailability issues. This axial code consists of two open codes: limited purchase channel and less variety.

- Limited Purchase Channel

Some participants pointed out that the situational barrier was linked to the lack of availability of green food products. For this reason, consumers needed to spend extra time to look for a place offering green food to purchase.

“Green food products are not available in every supermarket, and mainly the large or premium supermarkets have green food counters. Sometimes, I cease my thoughts of purchasing green food because of the inconvenience, distance and time.” (Participant 4)

- Less Variety

In addition, some interviewees reported that it was challenging to locate every variety of green food products in a supermarket, and only several representative products, such as vegetables, pulses, and tea, were identifiable.

“There are less varieties of green food products in the market, which limits me to find the one I want.” (Participant 25)

#### 3.4.3. Axial Code 3: Mistrust Issues

A quality attribute of green food belongs to confidence-based products; therefore, it becomes very necessary to increase consumers’ trust for establishing and promoting the growth of the green food market. However, mistrust issues and suspicious attitudes among Chinese consumers about eco-labelling food products are commonplace because of frequent food quality and safety incidents [6]. Consumers’ confidence is largely determined by their beliefs about the trustworthiness of food chain actors [65]. Specifically, some consumers do not trust the process of producing, manufacturing, labelling, and certification of green food products [18,66,67]. Mistrust issues are identified as one of the most salient factors hindering the IBG for consumers to buy green food in the Chinese context [18,68]. This axial code of mistrust issues includes three open codes: skepticism of producers, mistrust of certification, and negative social media reports.

- Skepticism of Producers

For mistrust barriers, some participants showed their skepticism of whether green food companies strictly control the production area, process, transportation, and storage. Participants found it difficult to verify if the available green product met green food standards.

“Although I have purchased green food before, I have always been skeptical of green food products. I worry about whether their planting and production meet the standards and whether the inspection is strict or not.” (Participant 7)

- Mistrust of Certification

Some interviewees expressed their reservations and even doubts about the certification and inspection organizations of green foods.

“I do not believe the certification system for green food, so I am not convinced whether green food is real or not. How can there be so many food safety problems if the green food certification authority is fully performing its duties?” (Participant 12)

- Negative Social Media Reports

Several interviewees reported that their information sources about green food were mainly from the internet or mass media. However, there were always some negative reports about green food being faked and not conforming to the standard on the internet, which significantly increased the interviewees’ concerns and worries.

“There are news exposing the problems of producing green food on the Internet, such as substandard and flashy food, which makes me very shocked and disappointed.” (Participant 19)

#### 3.4.4. Axial Code 4: Limited Knowledge

Consumers’ knowledge about green food is highly influenced by the information available on those products in the market. Some studies revealed that consumers had limited knowledge about the concept of eco-labelling products, and they did not know the background and benefits of those products [69,70]. Moreover, there are some similar ecological labels in the Chinese food market, such as green food, organic food, and hazard-free food. Because of the limited knowledge of those labels, consumers face difficulties in recognizing those eco-labels and distinguishing them from regular labels [71]. Limited knowledge about the concept, the benefits, how to recognize the labels, and identifying green food is acknowledged as one main barrier for consumers choosing green food products [6,72]. Hence, the axial code of limited knowledge consists of two open codes: benefit confusing and label confusing.

- Benefits Confusing

Some participants reported that they did not know much about the concept of green food, and its benefits.

“I know there are some benefits of green food products, but I think my knowledge is still limited about it. Thus, I become hesitant when I actually buy green food.” (Participant 5)

- Label and Brand Confusing

Some interviewees expressed that they found difficulties in distinguishing different labels. Besides, some labels were brand sensitive, and participants found it difficult to choose one reliable green food brand with high reputation, which led to an IBG.

“I recently found that there are some similar labels on the packages of different products, such as green food and organic food. I am very confused about these logos and do not know the differences among them. In addition, there are many green food brands in the market, but no particularly famous and well-known ones. It’s hard for me as a consumer to make a reliable choice when facing so much brands.” (Participant 1)

### 3.5. Impact of COVID-19 on Green Food Purchase Intention and the IBG

The impact of the COVID-19 crisis on consumers’ green food purchase intention and the IBG was investigated in the study. When participants were asked about the impact of COVID-19, the majority reported that the pandemic had a significant influence on their green food consumption. A probe question was raised to explore the influence of COVID-19 on their green food purchase intentions. In general, most of the participants perceived that the pandemic has increased their green food purchase intentions because of their growing health concerns. As a result, 46.43% of them reported that they have a heightened awareness of the importance of health and food safety. Consequently, participants are willing to invest money in their health and increase their percentage of green food purchases in their entire food purchases. A total of 28.58% of participants expressed that there was no impact from the COVID-19 crisis on their green food purchase intention behaviours, and they would purchase green food. However, 25.04% of respondents reported that the pandemic has decreased their intentions to purchase green food because of their worries about an income crisis, and they would reduce the percentages of buying expensive green food products. Table 4 shows the different perceived impacts of COVID-19 on participants’ green food purchase intentions.

Furthermore, another probing question was asked to investigate the effect of COVID-19 on the process of translating their green food purchase intentions to behaviours. The results are shown in Table 4. In general, 64.29% of participants reported that COVID-19 had a negative impact on this process and restricted their green food purchases for various reasons. In contrast, participants did express having high intentions to purchase green food products. Three common themes were observed from their responses: unavailability issues, price issues, and panic issues. For unavailability issues, some interviewees reported that, although they intended to buy green food, it was difficult to make purchases because of restrictions on going out and supply-side shocks during a pandemic.

“During the pandemic period, our family chose to stay at home and reduce going out. Although we wanted to buy green food, some shops and supermarkets were closed and there was no channel for making purchases. In addition, the supply of green food and amount of varieties available in supermarkets was less than before.” (Participant 12)

For pricing issues, some participants became more price-sensitive than in the past. Participants reported their income had decreased and they prevented themselves from purchasing more expensive items (green food).

“I began to be concerned about the price of food products during the pandemic. The prices of many food products were higher than before, as well as green food, which prevented me from purchasing green food and made me chose food with lower prices instead of higher priced items.” (Participant 3)

For panic issues, most participants indicated their fears and insecurities during the early outbreaks, which led to panic buying and hoarding behaviour, especially for key food items and convenience food (e.g., rice, flour, frozen foods, and instant noodles).

“I stocked up on lots of instant noodles, canned products, frozen dumplings, and other foods that could be kept for long-term storage. I did not consider to purchase green food at that moment.” (Participant 24)

However, 21.42% of respondents reported that COVID-19 had a positive impact on translating their green food purchase intentions to behaviours because of their health consciousness. Some participants believed consuming green food was necessary to improve their immunity and health, especially in this global health crisis period. When probing their purchase channels, all channels used online grocery delivery to purchase green food.

“Due to the COVID-19, I became increasingly careful about my health and my family’s health. Therefore, I choose to buy green food because of its high quality and high nutritional value. I used a mobile application to order green food products.” (Participant 9)

### 3.6. Action Diagram

**Figure 1:**
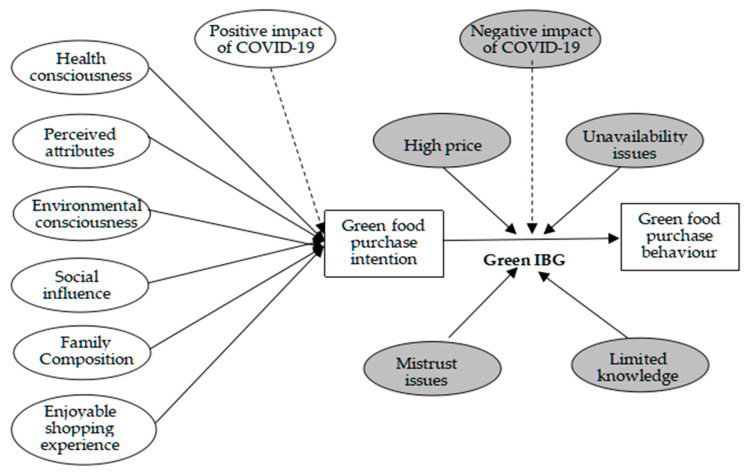
Factors influencing green food purchase intention and the green intention–behaviour gap (IBG). Note: The white ovals represent factors that drive green food purchase intentions. The grey ovals represent barrier factors that trigger green IBG. The solid line arrow represents a direct effect. The dotted line arrow represents an indirect effect.

Figure 1 represents the grounded theory framework that was deduced from the present research.

## 4. Discussion and Implications

During the interviews, researchers asked participants to describe the first three words that they think in terms of green food. ‘’Health’’ was the most frequent term mentioned by consumers, which is in agreement with the results from Ismael and Ploeger [44]. Our results also confirmed the findings from previous studies, in which “physical health” was one of the most strongly correlated terms with environmentally friendly food products [44,45,46,47]. In addition, participants agreed with other dimensions, including “intrinsic attributes” (e.g., sensory attributes, nutritional value, less pesticides), “extrinsic attributes” (e.g., high price, safe, sustainability, less pollution), and “psychological and personal aspects” (e.g., better life, enjoyment). Among these dimensions, “high price” and “safe” were the second and third most mentioned words after ‘’health’’. It can be explained that the economic and safe attributes of green food play significant roles in consumers’ green food perception, which also agrees with the findings from previous studies [6,25,60].

When probing factors that drive green food purchase intention, most consumers reported that they had the intention to buy green food because of their health consciousness, which is in line with our previous finding that “health’’ was perceived as the most correlated term of green food by consumers. Moreover, previous studies suggested that the factor of health benefits is the dominant driver behind environmentally friendly food intentional behaviour of consumers [6,73,74,75]. Therefore, marketers and green food providers should make health benefits the primary focal point when communicating with and convincing consumers to buy green food offerings. Moreover, consumers expressed that they perceived green food with specific attributes of being nutritious, tasty, natural, and of better quality, as well as good packaging. Thereby, these positive perceived attributes of green food products increased their intentions to choose green food. These findings underpin the assertion from most studies [6,76,77,78] that consumers’ purchase intentions and behaviours towards environmentally friendly food products are closely related to consumers’ perception of product-specific attributes. Marketers can highlight prominent attributes and benefits of green food products to consumers, and acknowledge the potentially detrimental effects of conventional food products. Environmental consciousness emerged as an important driver among consumers in this study, as consumers started to realize their responsibility in dealing with environmental and sustainablility issues. Purchasing green food was seen as pro-environmental behaviour, which can bring long-term benefits and provide better future orientations. This finding is consistent with the results from some studies discussing how environmental concerns have a direct and positive impact on the creation of consumers’ environmentally friendly food purchase intention [53,79,80]. Therefore, marketers and policymakers should properly communicate the environmental benefits to consumers. For example, the use of in-store digital advertisements to display how green food is grown and processed so the products can promote consumers’ senses of social responsibility for protecting and pursuing long-term sustainability. In addition, social influence was found to play a significant role in determining the creation of consumers’ green food intention in the investigation. Some interviewees reported that they preferred to follow a green lifestyle, similar to some bloggers and celebrities, which is consistent with the findings from Sogari et al. [81]. Therefore, marketers should pay more attention to celebrity and social media platform endorsements to positively guide green food consumption. The results also validate the findings from Qi and Ploeger [82], in which consumers with a Chinese cultural background were inclined to conform similarly with their reference groups, and focused more on their social status, which has been strongly predominant in their intentional green food purchases. Hence, marketers should emphasize the importance of interpersonal interactions and portray green consumers as high-status consumers through future marketing strategies. Furthermore, consumers’ family structures were observed to hold a large degree of importance in their green food intentions, as they had more willingness to buy for their children and elders, which supports the opinions from Liu et al. [6] and Zhang and Wu [59]. However, others studies reported that household and family structures did not have a significant impact [83,84], especially the study from Annunziata et al. [85], which showed that increasing household size reduces the likelihood of buying local and organic products in Italy. This disparity may be due to different family planning policies in different countries, as the majority of couples have one or two children in China. Thus, marketers should integrate actual conditions in China to develop and produce special products for target groups, and highlight their characteristics on the packaging to attract consumers’ interests. Some participants reported that positive shopping experiences motivated them to generate green food buying intentions. Thus, green food companies should intensify training for their salesmen and understand the important role of sales employees in creating valuable customers relationships and satisfaction. Additionally, green food stores or green food counters should provide customers with pleasant, comfortable, and high-end surroundings and professional services. Interestingly, our study discovered that promotional activity is another significant driving factor, whereas these findings do not yield results similar to previous studies from Ngobo [86] and Van Doorn and Verhoef [87], who reported a negative impact during environmentally friendly food consumption. A possible explanation is that most consumers know the premium attributes of green food product quality and are willing to purchase green food when sales promotions are attractive. Therefore, marketers should make proper promotion strategies to attract new customers and provide rewards to existing customers.

Moreover, most of the consumers presented positive attitudes towards green food in this study, whereas the results indicate that consumers’ actual purchase behaviour fell short of their purchase intentions to buy green food. Therefore, their purchase behaviour was not univocally consistent with attitudes and intentions, thus presenting the prevalent existence of the IBG phenomena in consumers’ green food consumption. These findings demonstrate that green or organic food markets are still suffering this disparity between favourable intention towards sustainable behaviour, which supports the results from Aschemann-Witzel and Zielke [88], Ismael and Ploeger [44], and Vermeir and Verbeke [89]. When probing the IBG of consumers making purchasing decisions of green food products, high price, unavailability, mistrust issues, and limited knowledge were identified as major factors contributing to this gap. This is consistent with the study of an attitude–intention–behaviour gap in green consumption from ElHaffar et al. [36]. Hence, these factors show a similarly important impact of the green gap despite the diversity of green products or green behaviours in different contexts. Specifically, a high price was the most significant factor in preventing consumers from transforming their intentions into buying more expensive green food products, which is widely confirmed by previous studies showing that a premium price of environmentally friendly food products has the strongest negative effect on a consumers’ final purchase [21,25,44,60,61]. While facing this high price gap, it is suggested that the implication for the green food sector is to engage in further efforts to reduce the high price image of green food as well as improve the product differentiation of the green food products. Meanwhile, the government should make relevant policies and strategies to reduce the prices of green food products and make these products more attractive and affordable to consumers. For instance, the government can issue preferential policies of monetary aids and tax cuts for relevant enterprises engaged in the production, processing, and sales of green food, particularly for enterprises in a transition period from conventional products to green products. In addition, there are many links and costs regarding traditional green food marketing channel patterns (e.g., agents–wholesalers–retailers), which leads to higher prices of green food products. Therefore, marketers and producers can apply smart supply chains, scientific procurement management systems, and new network marketing channel strategies to reduce their costs and improve their effectiveness. Moreover, these results confirm that perceived unavailability issues indeed acted as one factor in triggering a gap in green food consumption, which is in agreement with the results from other studies [25,44,63]. Although the green food market has existed for decades in China, it is still in the exploration and development stages, and there remains a sizable gap compared with fully developed, mature environmentally friendly food markets (e.g., organic markets in Germany). Thus, increasing the supply and variety of green food products in regular supermarkets and local community shops is an effective strategy to provide convenient availability and better selections. Online sale is a good strategy to widen the availability of green food, thus marketers should take full advantage of the internet to successfully expand green food consumption. Furthermore, trust crises have been mentioned as another main factor in many previous studies [6,10,21,82,90] as well as in this study. Therefore, effective solutions should extinguish the severe mistrust issues among Chinese consumers. To increase the degree of consumers’ trust in green food products, the government initially should increase their political functions, increase efforts in supervision, and establish strict laws and regulations to ensure sound standards and quality control of green food products. Marketers can properly communicate information about quality assurance to consumers using labels and certifications from government-approved third-party agencies to create awareness and compete for consumer trust. Limited knowledge was identified as an important obstacle for reducing consumers’ green purchases, which is also confirmed by previous studies [6,72]. Hence, it is essential to educate and inform consumers about the characteristics of green food products, the certification process, the differences between green food products and other products, and all other features that could be significant for consumers to accept these products and make rational purchasing decisions. Marketers can apply modern approaches to conduct educational promotions, such as posting promotional videos on popular media platforms (e.g., WeChat and TikTok). Additionally, while there are still no well-known green food brands (e.g., organic brand ALNATURA in the European market) that exist in the Chinese domestic market, breeding brand name green food products should be taken seriously to promote green food markets.

Regarding the impact of COVID-19, approximately two-thirds of consumers reported that the pandemic has increased their green food purchase intentions because of their growing health concerns. This is in agreement with results from Ben Hassen et al. [33] and Xie et al. [28], in that the COVID-19 crisis influenced respondents’ perceptions of health and risk, which in turn changed consumers’ sensitivity and beliefs and resulted in a further increase in organic food consumption. However, these increasing intentions did not increase the final amount of green food purchases. Green food purchases have reduced especially during the outbreak of COVID-19, despite having high purchase intentions, mainly because of issues of unavailability, price, and panic. In the early stages of the global pandemic, food supply chains were disrupted as a result of labour shortages, movement restrictions, and disruptions to transportation networks. Meanwhile, the majority of people complied to stay at home to reduce the possibility of spreading infectious disease, and many supermarkets and shops shortened their business hours to limit the spread of COVID-19 cases. Thus, the unavailability problem was significant to limit consumers’ green food purchases. Additionally, some respondents indicated that the pandemic had brought the individuals several negative psychological effects, which is consistent with findings specifically in a Chinese context [91]. In terms of food consumption, some consumers had become more price-sensitive, and their demand for income elastic products (e.g., green food) sharply declined as a result of a loss of household income and panic buying during the pandemic. Therefore, faced with increasing willingness and existing challenges, the green food industry should initially launch a comprehensive investigation and build practical measures of supply and demand problems that are influenced by the pandemic. If a second wave of the virus is experienced, adjusts to the initial demand shock, building greater supply chain resilience against potential supply-side shocks, and preventing the upswing in prices are key for the green food industry to ensure the availability and affordability for consumer green food purchases. Specifically, online shopping and grocery delivery services should be expanded and cooperate with famous e-commerce platforms (e.g., Taobao, Jingdong) to produce low-cost pricing strategies, enhance distribution channels, and strengthen promotional abilities, in order to enhance their competitiveness in the food market. Furthermore, this study’s findings can provide some theoretical implications for existing prevalent models whose constructs are easily influenced by the outcomes from the pandemic, such as the theory of planned behaviour (TPB) [9]. Hence, researchers should consider intervention efficacy or moderating effects of the outcomes of COVID-19 on the path in these related models. Even though COVID-19 may end in future, the impact of the COVID-19 factor can still be referred to in some similar events and factors.

## 5. Conclusions

The present qualitative study identifies the underlying influences on consumers’ green food consumption from the intention generation phase to intention execution phase from the perspectives of purchase intention and the IBG in the Chinese context. Specifically, during the intention generation phase, the study reports health consciousness, perceived attributes, environmental consciousness, social influence, family structure, and enjoyable shopping experiences as significant factors in driving green food intentional purchases among consumers. During the intention execution phase, high prices of green food, unavailability issues, mistrust issues, and limited knowledge were identified as salient factors of preventing consumers in translating their intention into green food purchase behaviour. Moreover, this work is one of the first studies to investigate the impacts from the COVID-19 pandemic on consumers’ purchase behaviour of green food products, which has not only revealed the existing problems, but also potentially mapped a positive future for the green food market in China. The above findings can enhance stakeholders’ understandings of the underlying facts and issues of consumers’ green food purchases. Thus, these findings can contribute to the design of future policy and industrial actions to better promote green consumption in the Chinese context. Despite the contributions of this study, some limitations are worth mentioning for future research goals. First, this study was based on an interview-based qualitative method, limiting the number of participants. An online recruitment approach was applied, so people without internet access were excluded, thus the findings cannot be considered to be representative of a significant population. Future research should focus on more diverse groups of populations from different backgrounds to view broader generalizations from the investigation. Additionally, this research measured green food purchases in general, whereas a previous study reported that different food products could be determined by distinct factors [92]. Therefore, a particular product segment or comparing a different segment of products is suggested for future research in order to make proper strategies for further marketing segmentation. Furthermore, some influencing factors with specific Chinese characteristics (e.g., group conformity, gain status) were identified in this study; hence, future research can apply a quantitative survey with a large sample to empirically validate the significant roles of the factors in Chinese food markets. Finally, because the impact of the COVID-19 pandemic on green food consumption may shift owing to uncertainties in its lifespan, tracking research with specific data analysis in the aftermath of COVID-19 is suggested for providing a more confident basis for responding to the influence of COVID-19 on green food consumption.

## Figures and Tables

**Table 1:** Characteristics of participants interviewed (*n* = 28).

Demographic Variables		Frequency	Percent (%)
Gender	Male	12	42.9
	Female	16	57.1
Age	<30	10	35.7
	30–50	11	39.3
	>50	7	25.0
Marital Status	Married with child or children	15	53.6
	Married	6	21.4
	Single	6	21.4
	Other	1	3.6
Education	Junior school or below	4	14.3
	High school or technical secondary school	9	32.1
	University or above	15	53.6
Monthly Income (RMB)	<4500	6	21.4
	4500–9000	14	50.0
	>9000	8	28.6
Whether there are elderly people over 60 or children under 12 in your home	YES	20	71.4
	NO	8	28.6

**Table 2:** Frequency of mention of individual words in four categories.

Categories	Individual Words	Number of Mentions
Intrinsic attributes of green food	Tasty	6
	Pesticide-free or less pesticide	4
	Nutritious	4
	Natural	2
	Additive-free or less additive	2
	Non-genetically modified organism (Non-GMO)	2
Extrinsic attributes of green food	High price	10
	Safe	8
	High quality	5
	Good packaging	3
	Environmentally friendly	3
	Clean	2
	Certified	2
	Sustainability	2
	Ecological	2
	Less pollution Untrustworthy Less variety	2 2 2
Physical health	Healthy	16
Psychological and personal aspects	Better life	3
	Enjoyment	2

Note: the individual words are the words participants were asked to write down as the first three words that come to their minds regarding green food.

**Table 3:** The number of participants in three consumer groups based on their green food purchase behaviour and intention.

Green Food Buyer Groups	Number of Study Samples (*n* = 28)	
	Purchase Behaviour	Purchase Intention
Regular ^1^	8	17
Irregular ^2^	4	5
Casual ^3^	16	6

^1^ Regular buyer refers to a consumer who buys/intends to buy green food more than other types of food. ^2^ Irregular buyer refers to a consumer who buys/intends to buy green food equally as other types of food. ^3^ Casual buyer refers to a consumer who buys/intends to buy green food less than other types of food.

**Table 4:** The impact of coronavirus disease 2019 (COVID-19) on participants’ green food consumption.

Perceived Impact	Number of Study Samples (*n* = 28)	
	Purchase Intention	Between Purchase Intention to Behaviour
Positive Impact	13	6
Negative Impact	7	18
No Impact	8	4
